# Optimized Enzymatic Synthesis of Hesperidin Fatty Acid Esters in a Two-Phase System Containing Ionic Liquid

**DOI:** 10.3390/molecules16087171

**Published:** 2011-08-23

**Authors:** Maria Elisa Melo Branco de Araújo, Fabiano Jares Contesini, Yollanda Edwirges Moreira Franco, Alexandra C.H. Frankland Sawaya, Thiago Grando Alberto, Natália Dalfré, Patrícia de Oliveira Carvalho

**Affiliations:** 1 São Francisco University, São Francisco de Assis Av., 218, Bragança Paulista, SP 12916-900, Brazil; 2 Laboratory of Food Biochemistry, Department of Food Science, Faculty of Food Engineering, State University of Campinas (UNICAMP), Monteiro Lobato Street, 80, Campinas 13083-862, Brazil; 3 Department of Plant Biology, Institute of Biology, State University of Campinas (UNICAMP), Campinas 13083-970, Brazil

**Keywords:** *Candida antarctica*, lipase, hesperidin, acylation

## Abstract

Response surface methodology (RSM) based on a five-level, three-variable central composite design (CCD) was employed for modeling and optimizing the conversion yield of the enzymatic acylation of hesperidin with decanoic acid using immobilized *Candida antarctica* lipase B (CALB) in a two-phase system containing[bmim]BF_4_. The three variables studied (molar ratio of hesperidin to decanoic acid, [bmim]BF_4_/acetone ratio and lipase concentration) significantly affected the conversion yield of acylated hesperidin derivative. Verification experiments confirmed the validity of the predicted model. The lipase showed higher conversion degree in a two-phase system using [bmim]BF_4_ and acetone compared to that in pure acetone. Under the optimal reaction conditions carried out in a single-step biocatalytic process when the water content was kept lower than 200 ppm, the maximum acylation yield was 53.6%.

## 1. Introduction

Flavonoids comprise a widely distributed group of polyphenolic plant secondary metabolites. They are widely used in food, cosmetics, and various other commodity preparations [1,2,3]. The biological, pharmacological, and medicinal properties of ﬂavonoids have been reviewed extensively [4,5]. Most of the beneficial health effects of flavonoids are attributed to their antioxidant activity, which is related to their ability to reduce free radical formation and scavenge free radicals [3]. However, the biological activity of these compounds seems to depend on their degree of lipophilicity, which controls their ability to reach the true site of free radical attack, inﬂuencing their interaction with particular cell types, proteins and enzymes [6,7]. Thus, the use of ﬂavonoids in several domains is limited by their low stability and solubility in both polar and non-polar media [8]. 

Hesperidin (6''-*O*-(α-L-rahmnopyranosyl)-D-glucose ﬂavonoid), a member of the flavanone group of flavonoids, is an abundant and inexpensive by-product of citrus cultivation widely available in Brazil. This compound can be isolated in large amounts from the rinds of some citrus species [e.g., *Citrus sinensis* L. (sweet orange), *Citrus aurantium* L. (bitter orange) and *Citrus unshiu* Marcov. (Satsuma mandarin) and has been reported to have antiallergenic, anticarcinogenic, antihypotensive, antimicrobial, and vasodilator properties [9].

A solution to improve the hydrophobic nature (lipophilization) of hesperidin, as well as other flavonoids, is their acylation, which can be accomplished by chemical or enzymatic processes. The chemical acylation of ﬂavonoids by various fatty acids has been patented, but this process is not regioselective and many of the hydroxyl groups present on the ﬂavan skeleton and on the sugar moieties can be esteriﬁed [10], which can lead to the acylation of some phenol groups that are directly implicated in the antioxidant activity of these molecules [10,11]. On the other hand, the enzymatic acylation of ﬂavonoids by lipases with fatty acids is more regioselective than chemical acylation and may enhance not only their solubility in various media, but also their stability and their antioxidant activity [12,13]. 

Ionic liquids (ILs, organic salts consisting only of ions, liquid at room temperature) have received growing attention as an alternative to organic solvents for the enzymatic transformation of various compounds. Their potential as reaction media for chemical and biocatalytic reactions arises from their speciﬁc physicochemical characteristics, such as lack of vapor pressure, thermal stability and properties related to hydrophobicity, polarity and good solubility for many polar or less polar organic compounds [14,15,16,17]. Employing ILs as media in these acylation reactions could allow for greater dissolution of ﬂavonoid substrates and reﬂect favourably on the productivity of the reaction system. 

**Scheme 1:**
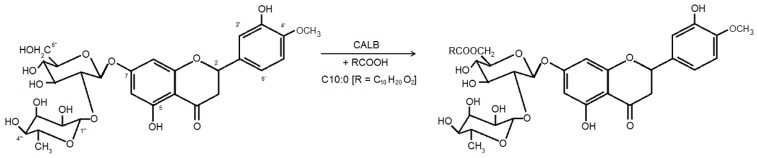
Enzymatic esterification of hesperidin with decanoic acid by immobilized *Candida antarctica* lipase B (CALB).

In the present work, the use of ionic liquids as media for the enzymatic synthesis of an acylated derivative of hesperidin (Scheme 1) has been described for the first time. The enzymatic acylation, through esterification, of a flavonoid disaccharide (hesperidin) using as acyl donor a medium chain fatty acid (decanoic acid) catalyzed by the well-known immobilized *Candida antarctica* lipase B (CALB) in a two-phase system containing [bmim]BF_4_ was used as a model reaction in order to investigate the influence of variables that affect the conversion yield (molar ratio of hesperidin to decanoic acid, [bmim]BF_4_/acetone ratio and lipase concentration). A statistical method that uses response surface methodology (RSM) is presented to indicate the parameter for an optimized synthesis process.

## 2. Results and Discussion

### 2.1. Influence of the Reaction Time and Hydration State

**Figure 1:**
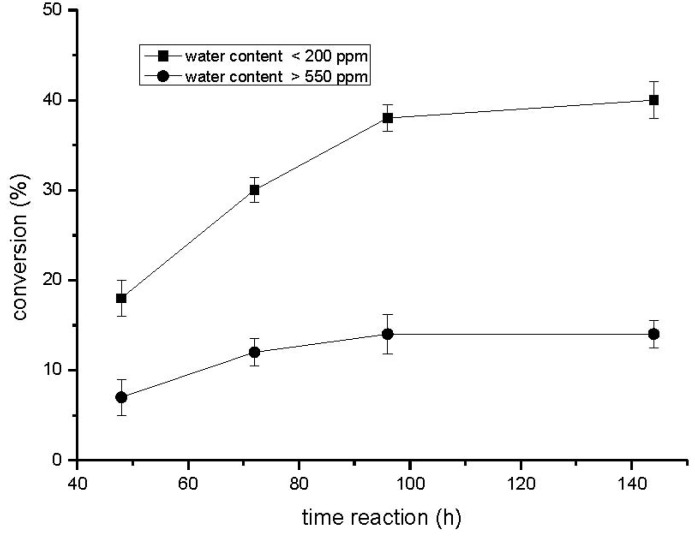
Reaction progress of the enzymatic acylation of hesperidin (100 mM) with decanoic acid (500 mM) catalyzed by immobilized CALB (8.0 mg/mL) in [bmim]BF_4_ and acetone (5:5, v/v) at 50 °C.

The results illustrated in the Figure 1 show that the conversion yields of acylated hesperidin are highest after 96 hs of reaction and slowly reach a plateau corresponding to the thermodynamic equilibrium. Water content was also important, because its increase led to an appreciable decrease in the amount of ester formed. Thus, the conversion yield decreases from 39.7% to 14.0% when the water content in the reaction medium increases from <200 ppm to higher than 550 ppm. It is important to highlight that the water was not added to the reaction system, it was controled only by the addition of the activated molecular sieves (4 Å) at two different reaction times (50 and 80 h). 

The results are in accordance with the literature. In the study of Katsoura *et al.* [18] the conversion yield for the acylation of naringin with vinyl butyrate catalyzed by immobilized *C. antarctica* lipase B in [bmim]BF_4_ or [bmim]PF_6_ based media decreased as the water content of the IL increased. While the conversion achived in 96 h of reaction in [bmim]BF_4_ was 61.6% with the water content as 0.1 (wt.%), the conversion was almost half of that value (37.7%) when the water content was 2.0.

This decrease of the conversion yield with the increase of the amount of water has been attributed to transport limitations of hydrophobic substrates from the solvent through the water layer surrounding the enzyme [19,20]. Added to that, the ionic liquids can dissociate into individual ions that can interact with the enzyme and/or the substrate and product molecules [20].

### 2.2. Optimization of the Esterification Process Using Response Surface Methodology

The variables and levels evaluated in this studied are shown in Table 1 and Table 2. A considerable variation in the results of the esterification of hesperidin catalyzed by CALB can be observed, indicating that the independent variables and their levels are very influential in the process (Table 2). The highest conversion value (55.3%) was obtained in run 6, using a molar ratio of the substrate hesperidin to decanoic acid of 1:7, a [bmim]BF_4_/acetone ratio of 8:2 (v/v) and 11.6 mg/mL of lipase. On the other hand, the lowest conversion obtained was 23.1% (run 9) when using a molar ratio of the substrates of 1:1, a ratio of [bmim]BF_4_/acetone of 5:5 (v/v) and 8.0 mg/mL of lipase. 

The regression coefficients are shown in Table 3. The effects of the three factors were considered to be statistically significant (p < 0.1) at the 90% confidence level. The coefficients selected for the linear effects were the molar ratio of substrates, the molar ratio of solvents and lipase concentration and the quadratic effect of the molar ratio of substrates. The interactive effects between the molar ratio of substrates and the molar ratio of solvents were also significant. The analysis of variance (ANOVA) gave the following regression equation (in terms of the coded factors, Equation 3).

Y = 39.99 + 4.70·X1 − 3.22·(X1)^2^− 5.79·X2 + 5.71·X3 − 2.90·X1·X2 (3)

where Y was the conversion (%) (response variable) and X1, X2 and X3 are the coded values of the independent variables: molar ratio of hesperidin to decanoic acid, [bmim]BF_4_/acetone ratio and lipase concentration, respectively. The R^2^ value (0.8855) indicates the accurancy of the model and provides a measure of how much variability in the observed response values can be explained by the experimental factors and their interactions. The statistical significance of the second-order model equation was evaluated by the F-test analysis of variance, which showed that this regression was statistically significant (p < 0.1) at the 90% confidence level (Table 4).

**Table 1:** Variable and levels for central composite design in the enzymatic acylation of hesperidin with decanoic acid catalyzed by immobilized lipase from *Candida antarctica*.

Variable		Coded variable levels				
		−1.68	−1	0	1	1.68
Molar ratio of hesperidin to decanoic acid	X_1_	1:1	1:3	1:5	1:7	1:9
[bmim]BF_4_/acetone ratio (v/v)	X_2_	10:0	8:2	5:5	2:8	0:10
Lipase concentration (mg/mL)	X_3_	2.0	4.4	8.0	11.6	14.0

**Table 2:** Central composite design and responses in the enzymatic acylation of hesperidin with decanoic acid after 96 h of reaction.

Run	Coded variable levels			Observed conversion yield ^a^ (%)	Predicted conversion yield ^a^ (%)
	X_1_	X_2_	X_3_		
1	−1	−1	−1	29.2	29.25
2	+1	−1	−1	47.0	44.45
3	−1	+1	−1	28.2	23.47
4	+1	+1	−1	23.7	27.07
5	−1	−1	+1	42.5	40.67
6	+1	−1	+1	55.3	55.87
7	−1	+1	+1	27.2	34.89
8	+1	+1	+1	38.3	38.49
9	−1.68	0	0	23.1	23.01
10	+1.68	0	0	39,2	38.79
11	0	−1.68	0	48.5	49.72
12	0	+1.68	0	35.1	30.26
13	0	0	−1.68	24.3	30.39
14	0	0	+1.68	50.2	49.58
15	0	0	0	42.0	39.99
16	0	0	0	38.6	39.99
17	0	0	0	39.5	39.99
18	0	0	0	43.3	39.99

^a^ Conversion refers to percentage of the total ester formed.

**Table 3:** Regression coefficients for lipase activity from the central composite design after 96 h of reaction.

	Regression	Standard Error	t(8)	p	−90,%	+90,%
Mean/Interc. *	40.8465	2.06081	19.8205	0.00000	36.0943	45.5988
X1 ^a^(L)(Linear) *	4.7039	1.11755	4.2091	0.00296	2.1268	7.2810
X1(Q)(Quadratic) *	−3.3864	1.16242	−2.9132	0.01949	−6.0670	−0.7059
X2 ^b^ (L) *	−5.7903	1.11755	−5.1812	0.00084	−8.3674	−3.2132
X2 (Q)	0.2983	1.16242	0.2566	0.80393	−2.3822	2.9788
X3 ^c^ (L) *	5.7100	1.11755	5.1093	0.00091	3.1329	8.2871
X3 (Q)	−1.2606	1.16242	−1.0844	0.30974	−3.9411	1.4199
X1L by X2L *	−2.9000	1.45951	−1.9869	0.08215	−6.2656	0.4656
X1L by X3L	1.3750	1.45951	0.9420	0.37372	−1.9906	4.7406
X2L by X3L	−0.9500	1.45951	−0.6509	0.53335	−4.3156	2.4156

* Statistically significant effects. ^a^ Molar ratio of hesperidin to decanoic acid; ^b^ [bmim]BF_4_/acetone ratio (v/v); ^c^ Lipase concentration (mg/mL).

**Figure 2:**
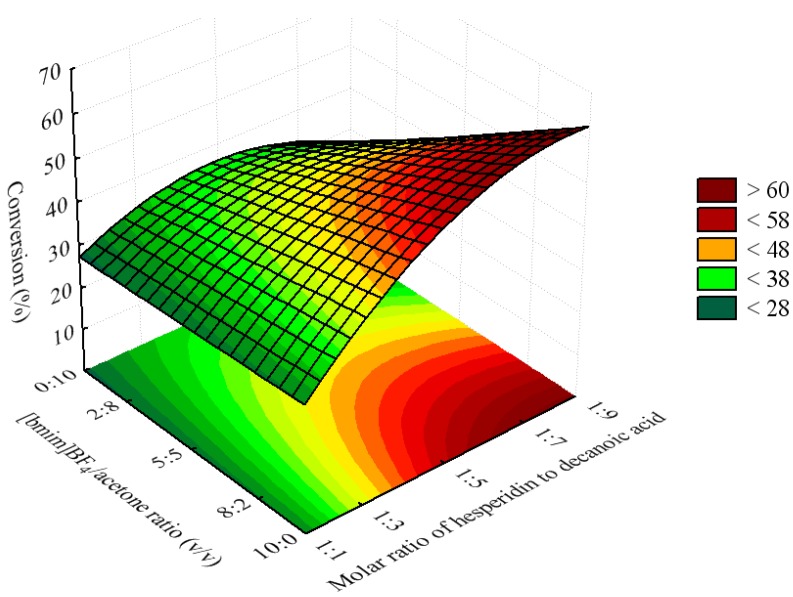
Response surface for the esterification of hesperidin with decanoic acid catalyzed by immobilized CALB (11.6 g/mL) at 50 °C for 96 h, as a function of the molar ratio of hesperidin to decanoic acid and [bmim]BF_4_/acetone ratio.

**Figure 3:**
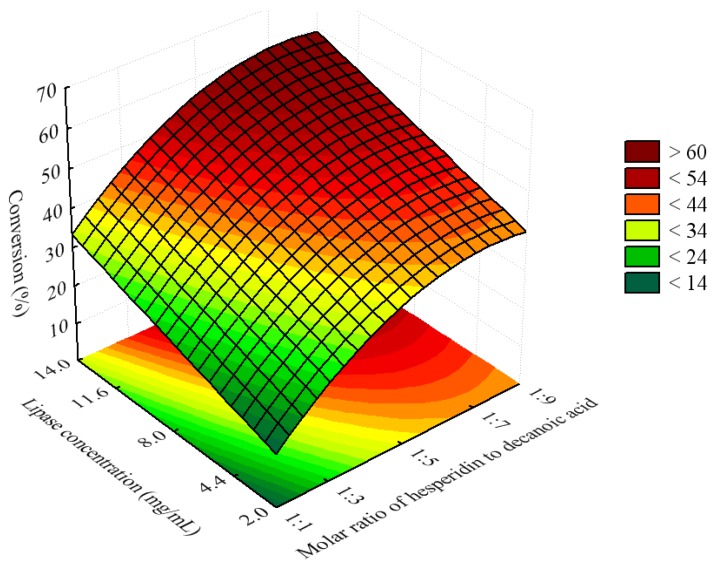
Response surface for the esterification of hesperidin with decanoic acid catalyzed by immobilized CALB at 50 °C for 96 h, as a function of the molar ratio of hesperidin to decanoic acid and lipase concentration. The ratio of [bmim]BF_4_/acetone was fixed as 8:2 (v/v).

**Figure 4:**
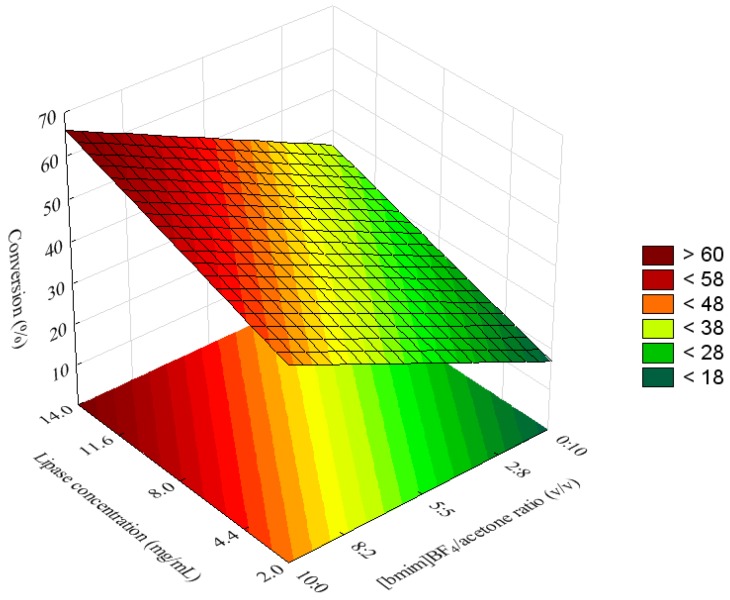
Response surface for esterification of hesperidin with decanoic acid catalyzed by immobilized CALB at 50 °C for 96 h, as a function of the molar ratio of [bmim]BF_4_/acetone and lipase concentration, hesperidin to decanoic acid and lipase concentration. The molar ratio of hesperidin to decanoic acidwas fixed as 1:7.

Upon comparison (Table 2), a strong correlation was observed between the predicted and experimental data. The behaviors of both were synchronized, though there were some variations according to the R^2^ value (0.8855). As can be seen in Figure 2, Figure 3 and Figure 4, the amount of esters formed during the acylation of hesperidin with decanoic acid increased when the molar ration of acyl donor to flavonoid was higher, and as the quantities of IL and the lipase were higher as well. Figure 2 shows the negative interactive effects between molar ratio of substrates and molar ratio of solvents. The surface indicates that higher conversion values can be obtained using higher amounts of dacanoic acid. However, if the experiment were carried out using only acetone as solvent (1.68, in terms of coded value) and using more than 1:5 of molar ratio of hesperidin to decanoic acid, then a decrease in the conversion yield would be observed. This is different behavior from what would be observed if the experiment were carried out using a higher ratio of [bmim]BF_4_/acetone, taking into account that it would not decrease the conversion, based on the surface (Figure 2).

**Table 4:** ANOVA for the central composite design after 96 h of reaction.

Source of variation	Sum of squares	Degrees of freedom	Mean square	F-value	p
Regression	1412	5	282.476	18.56	0.000028
Residual	183	12	15.21833		
Lack of fit	169	9	18.77778	4.14	
Pure error	14	3	4.54		
Total	1595	17			

Regression coefficient: R2 = 0.8855/F_0.1;5;12_ = 2,39.

The positive effect of the molar ratio of acyl donor to alcohol on the conversion yield has also been reported for the enzymatic acylation of sugars and glycosides in various organic media [21,22,23]. This effect could be attributed to a thermodynamic shift of the equilibrium in favor of the synthesis of hesperidin ester due to excess acyl donor (decanoic acid).

Kontogianni *et al.* [24] observed that the variation of the concentration of lipase CAL-B during the acylation of naringin in the presence of decanoic acid as an acyl donor and *tert*-butanol as a solvent, led to an increase of the conversion yield. However, a plateau was reached when lipase concentrations were higher than 15 g/L. The highest value of the conversion yield was about of 40% after 240 h of incubation. The specificity towards hesperidin can be attributed to the fact that the CALB exhibits specificity towards primary hydroxyl group on the glycosyl moiety alcohols of several flavonoids, as previously reported in the case of naringin [25].

### 2.3. Verification of the Optimal Conditions

Based on the regression equation and the response surfaces, the optimal conditions of the esterification are a molar ratio of hesperidin to decanoic acid of 1:9 (X1), [bmim]BF_4_/acetone ratio of 10:0 (v/v) (X2) and lipase concentration of 14.0 mg/mL (X3). However, taking into account that a small amount of acetone is important for the solubilization of the hesperidin and that the enzyme represents one of the highest costs of the process, the validation of the experimental model was carried out using X1 at 1:7, X2 at 8:2 and X3 at 11.6 mg/mL, at 50 °C, for 96 h of reaction. The conversion obtained was 53.6%, which is not statistically different from the predicted value (55.8%). This result is higher compared to that obtained (40%) in the experiment carried out prior to optimization when the molar ratio of substrates was 1:5, the ratio of the solvents was 5:5 (v/v) and the lipase concentration of 8.0 mg/mL. These results indicated a substantial improvement in the yield of the process, which is important from a commercial point of view. 

## 3. Experimental

### 3.1. General

Immobilized *Candida antarctica* 435 lipase (Novozym 435, EC 3.1.1.3, ≥10,000 U/g) was purchased from Novo Industries. Hesperidin, decanoic acid, [bmim]BF_4_ (1-butyl-3-methylimidazolium tetrafluoroborate) and acetone were purchased from Sigma and Merck and were of the highest available purity. Molecular sieves (4 Å, 8–12 mesh beads) were purchased from Sigma.

### 3.2. Enzymatic Acylation Procedure

The enzymatic reaction was carried out in screw-capped glass tubes using [bmim]BF_4_ and/or acetone as solvent. Hesperidin (100 mM) and decanoic acid (adjusted to different molar ratios) were solubilized in solvent (10 mL). The acylation was started by the addition of immobilized *Candida antarctica* lipase (2.0–14.0 mg/mL) and the mixture was incubated at 50 °C with agitation on an orbital shaker (150 rpm) for up to a maximum of 144 h. The water content of the reaction medium was determined by a coulometric Karl Fisher apparatus (KF 737II coulometer). Activated molecular sieves (4 Å) were added at 100 g/L after certain times of reaction (50 and 80 h) to control the water content in the reaction medium. Finally, the reaction was stopped and the enzyme was filtered off. Bioconversion was monitored periodically (48, 72, 96 and 144 h) by UPLC-MS to determine ester production, with bioconversion (%) calculated following UPLC analysis as the area of the ester product divided by the total area, multiplied by 100.

### 3.3. Experimental Design

In order to further investigate the conversion yield of hesperidin acylated derivative in the reaction system composed of 50% of [bmim]BF_4_ and 50% acetone, two selected factors, including incubation time and the hydration state of the reaction media, were examined using the concentration of enzyme as 8.0 mg/mL and the molar ratio of hesperidin to decanoic acid as 1:5, respectively. Following that, a 2^3^five level, three-variable central composite design (2^3^–CCD) was adopted for the optimization of the acylation reaction. The CCD was used to study the effect of the independent variables molar ratio of hesperidin to decanoic acid (1:1–1:9), [bmim]BF_4_/acetone ratio (10:0–0:10, v/v) and lipase concentration (2.0–14.0 mg/mL). The dependent variable is the conversion (%) of hesperidin fatty acid esters. 

The variables were coded according to Equation (1):

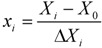
(1)
where x_i_ is the dimensionless coded value of an independent variable, X_i_ is the real value of an independent variable, X_0_ is the real value of an independent variable (X_i_) at the center point and ΔX_i_ is the step change value. The variables and levels are shown in Table 1 and Table 2. The system behavior was determined by a second-order polynomial equation, based on the equation below:

Y = β_0_ + Σβ_i_x_i_ + Σβ_ii_x_i_^2^ + Σβ_ij_x_i_x_j_ (2)

where Y is the predicted value for the response, β_0_ is the offset term, β_i_ is the linear effect coefficient, β_ii_ is the squared effect coefficient and β_ij_ is the interaction effect. x_i_x_j_ represents the interaction between different coded values, where i is one parameter and j is other.

### 3.4. Statistical Analysis

The 2^3^–CCD was defined by Statistic 7.0 software (Statsoft, Inc., Tulsa, OK, USA) for three factors with a total of 18 assays, six axial points (α) and four replicates at the center point to estimate the experimental error and to investigate the suitability of the proposed model. Student's t-tests were used to determine the statistical significance of the regression coefficients. Significance of data was tested using analysis of variance (ANOVA) statistical test. Variables with a confidence level greater than 90% were considered to have a significant influence on conversion yield (%). The time of reaction was not considered a significant variable in this experimental design, since the experiments were accomplished at five different time frames in order to study the reaction kinetics.

### 3.5. Validation of the Experimental Model

The experimental model was validated by carrying out the biocatalytic process with the statistically significant variables at what was considered their optimal concentrations. The result represents the average of three replicates.

### 3.6. UPLC-MS Quantification

The chromatographic separation was achieved using an Acquity UPLC system (Waters, Milford, MA, USA) equipped with a Waters UPLC BEH column (2.1 × 50 mm, 1.7 µm particle size) at a temperature of 25 °C, injecting 5 μL of each extract. A gradient was applied using two mobile phases—(A) purified water with 1% formic acid; and (B) acetonitrile with 1% formic acid—starting with 2% B, ramping to 35% B in 6 min, and then to 100% B from 6.10 to 6.50 min, and finally returning to the initial conditions. Detection was carried out in both the positive and negative ion modes using an Aqcuity TQD mass spectrometer with an ESI source (Micromass Waters, Milford, MA, USA) under the following conditions: capillary ± 3000 V, cone ± 30 volts, temperature 150 °C; ranging between 110–700 *m/z.* The m/z values of hesperidin, decanoic acid and decanoic acid monoester with hesperidin were 609.1, 172.3 and 763.4, respectively. The retention times of these compounds were 4.30, 8.13 and 7.84 minutes, respectively.

## 4. Conclusions

The acylation of hesperidin with decanoic acid as an acyl donor was dependent of the three variables studied in this work. The results showed that the best results were found using a molar ratio of hesperidin to decanoic acid of 1:7, [bmim]BF_4_/acetone ratio of 8:2 (v/v) and a lipase concentration of 11.6 mg/mL. As expected, higher amount of lipase and a higher ratio of acyl donor to the hesperidin resulted in higher conversion yields. However, the results indicated that better conversion values were achieved using higher amounts of ionic liquid than acetone. This is very interesting from the environmental point view considering that IL is a non-toxic solvent.
